# High-Resolution Magnetic Resonance Imaging of the Regenerating Adult Zebrafish Heart

**DOI:** 10.1038/s41598-017-03050-y

**Published:** 2017-06-07

**Authors:** Jana Koth, Mahon L. Maguire, Darryl McClymont, Leonie Diffley, Victoria L. Thornton, John Beech, Roger K. Patient, Paul R. Riley, Jürgen E. Schneider

**Affiliations:** 1 Weatherall Institute of Molecular Medicine, JR Hospital, Oxford University, Oxford, OX3 9DS UK; 2 Department of Physiology, Anatomy and Genetics, Oxford University, Oxford, OX1 3PT UK; 3 BHF Experimental MR Unit, Wellcome Trust Centre for Human Genetics, Oxford University, Oxford, OX3 7BN UK; 4 Department of Oncology, Oxford University, Oxford, OX3 7DQ UK

## Abstract

The adult zebrafish is a well-established model for studying heart regeneration, but due to its tissue opaqueness, repair has been primarily assessed using destructive histology, precluding repeated investigations of the same animal. We present a high-resolution, non-invasive *in vivo* magnetic resonance imaging (MRI) method incorporating a miniature respiratory and anaesthetic perfusion set-up for live adult zebrafish, allowing for visualization of scar formation and heart regeneration in the same animal over time at an isotropic 31 µm voxel resolution. To test the method, we compared well and poorly healing cardiac ventricles using a transgenic fish model that exhibits heat-shock (HS) inducible impaired heart regeneration. HS-treated groups revealed persistent scar tissue for 10 weeks, while control groups were healed after 4 weeks. Application of the advanced MRI technique allowed clear discrimination of levels of repair following cryo- and resection injury for several months. It further provides a novel tool for *in vivo* time-lapse imaging of adult fish for non-cardiac studies, as the method can be readily applied to image wound healing in other injured or diseased tissues, or to monitor tissue changes over time, thus expanding the range of questions that can be addressed in adult zebrafish and other small aquatic species.

## Introduction

Over the last decade, adult zebrafish have emerged as an invaluable model for vertebrate cardiac regeneration^1^, yet most studies to-date have relied on distinct time points from different individuals post-injury with conventional histology or immunohistochemistry on tissue sections as the primary read-out. This makes it impossible to determine the degree of regeneration, since the original extent of damage in the repairing region can only be assumed. Furthermore, the initial lesion size, or even the presence of an injury, varies greatly between different individuals. Despite these important considerations longitudinal high-resolution and non-invasive imaging of the same adult fish heart during the regeneration process has not yet been achieved.

Embryonic and larval zebrafish have been powerful models in basic science and biomedical research for several decades due to their external development and transparency, making them highly amenable for *in vivo* optical imaging. However, this advantage decreases rapidly after 2–3 weeks as tissues become denser and more difficult to image by light microscopy. Although pigment-free fish lines with transparent skin^2^ allow for imaging subcutaneous superficial cell layers, the tissue protein content and resulting opaqueness severely limit *in vivo* deep tissue imaging. In addition, the movement and high heart contraction rate (~130 beats per/min) is challenging for optical high-resolution imaging. Tomographic techniques such as Magnetic Resonance Imaging (MRI), Computed Tomography (CT), or 2D/3D cardiac ultrasound (i.e. echocardiography) provide non-invasive deep tissue imaging without relying on optical transparency^3, 4^ and can, therefore, be applied to the heart.

Echocardiography is an inexpensive and widely available technique, but frequently provides only one- or two-dimensional information and is limited to morphological and functional investigations. CT can provide excellent spatial resolution, but relies on ionizing radiation and the application of dedicated stains to achieve soft tissue contrast. MRI is well established in clinical diagnostics as it provides multi-parametric information on the heart radiation-free and non-invasively. The design of the measurement sequence (i.e. gradient echo, spin echo, etc.) and the choice of the corresponding experimental parameters (i.e. flip-angle of excitation radio-frequency (RF) pulse(s), echo time, repetition time etc.) utilise the difference in MR properties between tissue types and/or between healthy and diseased tissue, and thus achieve intrinsic contrast without the need for exogenous contrast agents. MRI is routinely used clinically not only to assess cardiac anatomy and function, but also to characterize the heart muscle at a tissue-level (for example the extent of injury after a myocardial infarction) in patients with heart disease. Furthermore, it can non-invasively provide structural and metabolic information. MRI is also frequently used preclinically for *in vivo* imaging in non-aquatic animal models of (cardiovascular) disease, including small (eg. rodents) and large (eg. sheep, pigs) mammals. In both settings, synchronizing the measurement with the heartbeat allows for minimizing motional influence and to obtain either a static or dynamic series of images (‘cine’) of the heart. *In vivo* MRI has also previously been applied to image early developmental stages of frog embryos, which, unlike transparent zebrafish embryos, are too opaque for optical imaging of deeper tissue layers due to their rich yolk content^5, 6^. However, presently there is an unmet need for deep tissue imaging in small species that are not optically transparent (and thus beyond the current abilities of light-microscopy), but not big enough to be imaged at sufficient resolution with readily available and established MRI, CT or Echocardiography techniques. The small size of adult zebrafish (cross-sectional diameter <5 mm) and in particular, the size of an adult zebrafish ventricle (~750 µm in length) requires adequate spatial resolution to reliably detect any tissue alterations. While 2D *in vivo* MRI of gross morphology in adult zebrafish has been previously reported^3^, the published resolution was insufficient to study small histological changes in the presented images.

In this study we combined cutting-edge technical developments in MRI, novel data processing approaches and 3D-printing to design a set-up and scan technique for resolving very small changes *in vivo* during zebrafish heart regeneration. 3D scans covering the thoracic region of the same adult zebrafish were acquired longitudinally at a 31 µm isotropic voxel resolution, a level of resolution which is typically only achieved *ex vivo* in fixed specimens. Using compressed sensing accelerated 3D scans^7, 8^ the internal anatomy of the whole zebrafish heart could be resolved within ~30 min acquisition time. Compressed Sensing (CS) is used in MRI to accelerate the data acquisition process, which is crucial for many imaging demands, in particular for *in vivo* imaging, where conventional high-resolution imaging may not be feasible within the available time frame ^7^. It is based on the assumption that the images are sparse (i.e. most of the energy is contained in a few elements, with the remaining elements close to zero) in a domain that is incoherent with the MRI acquisition, which means that the acquisition can be under-sampled and the missing samples may later be recovered using non-linear reconstruction techniques.

At present there are several cardiac injury techniques applied in zebrafish, each with their own advantages and disadvantages. For partial ventricular resection injury^1^ ~15% of the ventricular apex can be resected with fine scissors, allowing for a very fast (<1 min) procedure, after which a blot clot forms. For cryoinjury^9–11^, the pericardial sac is opened and the ventricle exposed as for resection injury, but instead of cutting away the tip of the apex, a cooled (eg. in liquid nitrogen) probe tip is used to freeze the tissue. For genetic ablation injury, cardiomyocytes can be inducibly and selectively ablated in a gene and tissue specific manner^12–14^. Cryo- and resection injury are localized to the apex of the heart, whereas genetic ablation is distributed throughout the targeted tissue type. The type of injury has implications for the amount and distribution of damaged or dead cardiomyocytes and thus on the appearance of the lesion and amount of scar tissue. Whereas the tissue removal leads to a blood clot and a small scar tissue area, cryoinjury leaves a large region of damaged or dead heart tissue leading to a larger scar region. The amount of scar tissue formation in the ablation model varies depending on the extent of injury, but is diffuse and limited to the targeted cell type and not all cardiac tissues. Thus, out of the three methods, cryoinjury resembles most a myocardial infarction situation found in patients or in LAD (left anterior descending artery) ligation injury in rodent models of cardiac infarction and myocardial ischemia. We have used cryo- and resection injury in our study as they are both distinctly localized to the apex of the ventricle.

The applicability of the MRI method was tested by investigating uninjured, sham-injured, cryo-injured and resection-injured Tg(*hsp70l:dnfgfr1-EGFP*) fish, which either show efficient heart repair when kept at normal temperatures, or impaired heart regeneration when exposed to a daily heat-shock at 38 °C. We demonstrate that the presented technique provides sufficient sensitivity to visualize the resolution of the lesion/scarring, and to distinguish between normal and impaired repair processes in both cryo- and resection injury. As such we demonstrate unequivocal evidence of the extent of initial injury and bona fide regeneration in a living individual animal over time. Importantly, this method is equally suitable for a broad range of 3D imaging applications in adult zebrafish beyond cardiac regeneration studies, and it has the potential to expand the experimental range of this model to address questions regarding organ function and repair that cannot be studied during embryonic and larval stages.

## Results

### MRI is suitable to visualize adult zebrafish cardiac morphology and injury *ex vivo*

**Figure 1:**
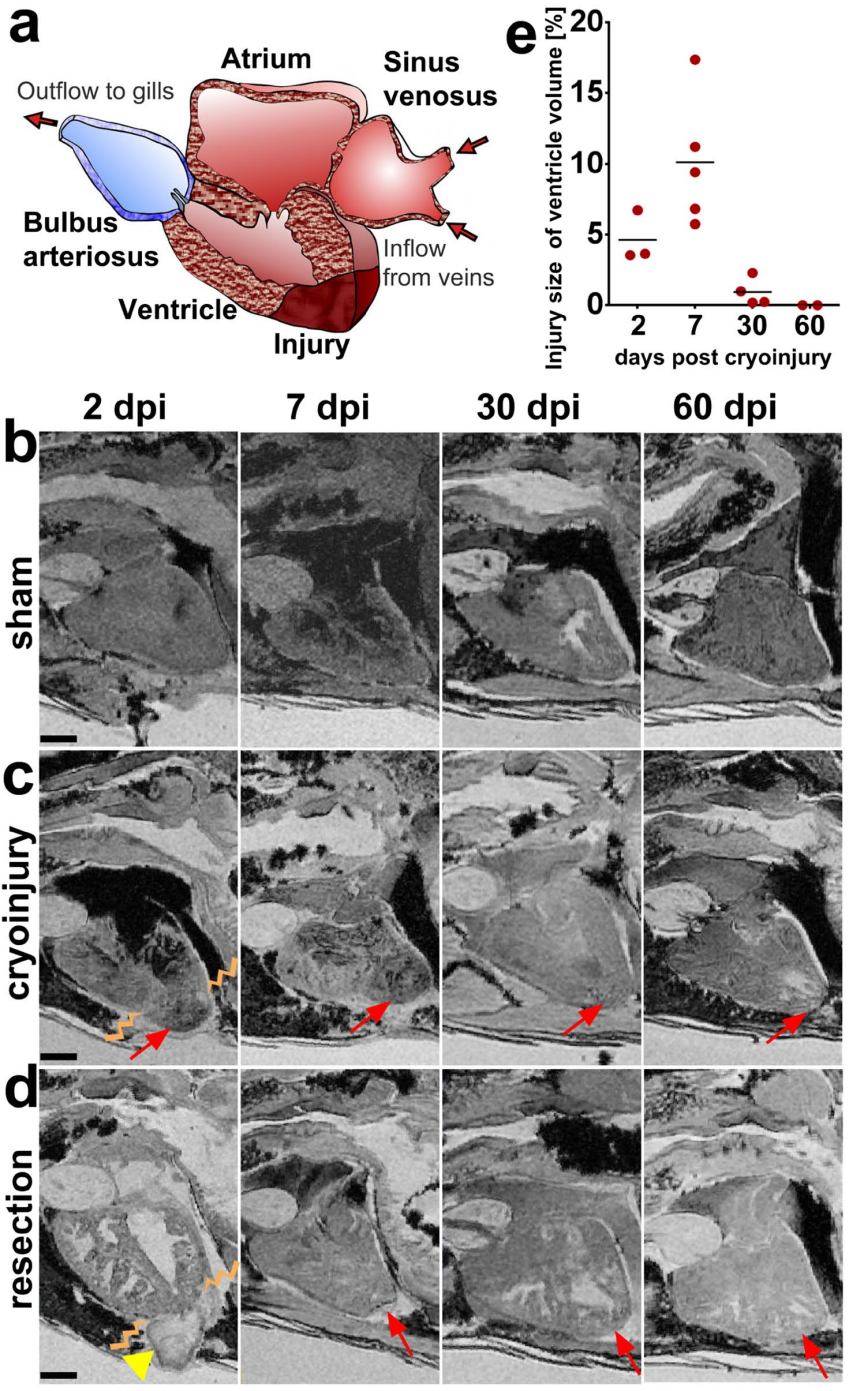
*Ex vivo* MRI of heart regeneration in different fixed specimens. **(a)** Schematic drawing of the zebrafish heart in sagittal section view as in the MR images below. The apex of the ventricle is drawn with a cryoinjury to illustrate the location of the lesions. **(b–d)** Optical sagittal MR sections showing the regeneration process in **(b)** sham operated controls, **(c)** cryo-injured, and **(d)** resected ventricles at 2, 7, 30, and 60 days post injury (dpi) in wt fish. The lesion is indicated with an arrow and the blood clot after resection is indicated with an arrow-head. The border between healthy and damaged heart tissue is indicated by zig-zag lines at 2 dpi. **(e)** Graph showing the cryoinjury volume in percentage of the ventricular volume at the 4 time points (see **c**) investigated for 13 individual fixed specimen [lesion size per ventricle (Y) and the mean value (bar line) at each post injury time point (X)] demonstrating the variability of the lesions between specimen. Anterior to the left, dorsal to the top. n/ time point = 2 per sham group and 4 per injury group. Scale bar 0.5 mm.

Prior to setting up *in vivo* imaging we tested the feasibility of MRI in detecting injuries to the cardiac ventricle with sufficient resolution in fixed specimens of a control group (no surgery, data not shown), sham operated (no injury to the ventricle but open pericardium), cryo-injured and resected ventricle fish groups, and imaged specimens fixed at 2, 7, 30 and 60 days post injury (dpi) (Fig. 1). Our aim was to acquire a very high resolution scan of fixed (*ex vivo*) specimens under each of these conditions to establish a baseline of what is achievable without motion or limitations in scan time. As this is the first study to present MRI of zebrafish heart repair, the *ex-vivo* images serve as a bench-mark against which to judge the quality of the *in vivo* MR images. Heart injuries were readily detectable via a contrast difference between injured and uninjured heart tissue in the optical sections of the 3D images of these fixed but intact specimens. An MR signal is determined by numerous factors, including the number of ^1^H nuclei (hydrogen atoms) present in a voxel, their relaxation properties and others. Therefore, similar grey levels within an image do not necessarily mean they represent the same tissue. In the sham hearts, there is no variation in appearance of the myocardial apex from that of the rest of the myocardium (Fig. 1b). However, as can be seen from Fig. 1c, the apex of the cryo-injured heart appears darker in the image than the remote myocardium, particularly at the 2 and 7 dpi time points. At later stages the cryo-injured regions appeared less dark when compared to healthy tissue, indicating progressive scar resolution. Conversely, the apex of the resection-injured myocardium appears bright in comparison to the remote uninjured myocardium (Fig. 1d). Some variation of contrast within an organ is to be expected, as the tissue is not perfectly uniform in composition and structure. For example, at the 60 dpi time point, the sham myocardium in Fig. 1b appears to have a speckled pattern of dark points spread uniformly over the myocardium. It does not, however, have a well-defined region of hypointensity that could reasonably correspond to an injury – rather the variation in contrast is diffuse and uniform. Assessment of the injury is, therefore, based on both image contrast and anatomy. Some regions of hypointensity are observed in similar images of fixed hearts of larger animals where blood has remained prior to fixation; the iron containing clotted blood (paramagnetic) causes negative contrast and signal voids, although speculation as to the origin of the occasional signal voids in the atria, or the heterogeneous appearance of some of the zebrafish hearts, is beyond the scope of this study. Identification of the damaged tissue was greatly facilitated by analysing the z-planes through each stack in orthogonal views, revealing the extent of the injury and the borders with healthy adjacent tissue. When reconstructing the ventricles and lesions in the acquired 3D images (using Amira software) and measuring the injury volumes, we noted variability in the injury size (~4%, ~10% and ~1% of total ventricle volume at 2, 7 and 30 dpi respectively), despite taking the utmost care to cause comparative lesions during cryoinjury (Fig. 1e). As the injury in each fish is carried out on a live beating ~750 μm ventricle by either manually cutting the apex or freezing of the apex with a cryoprobe, each injury is unique. Thus, there is variability between fish, which has not been adequately addressed in zebrafish heart repair studies to date, and which is a confounding factor when discussing and comparing repair rates deduced from two-dimensional images from single post-injury time points and via sections of different hearts/animals subjected to varying injury settings/drug treatments or interventions.

Collectively the data from the imaging of fixed specimens suggested the MRI resolution would indeed be suitable for longitudinal assessment of injury regression during zebrafish heart regeneration and we, therefore, proceeded to attempt equivalent resolution for live imaging.

### MRI is suitable to interrogate adult zebrafish cardiac morphology *in vivo*

**Figure 2:**
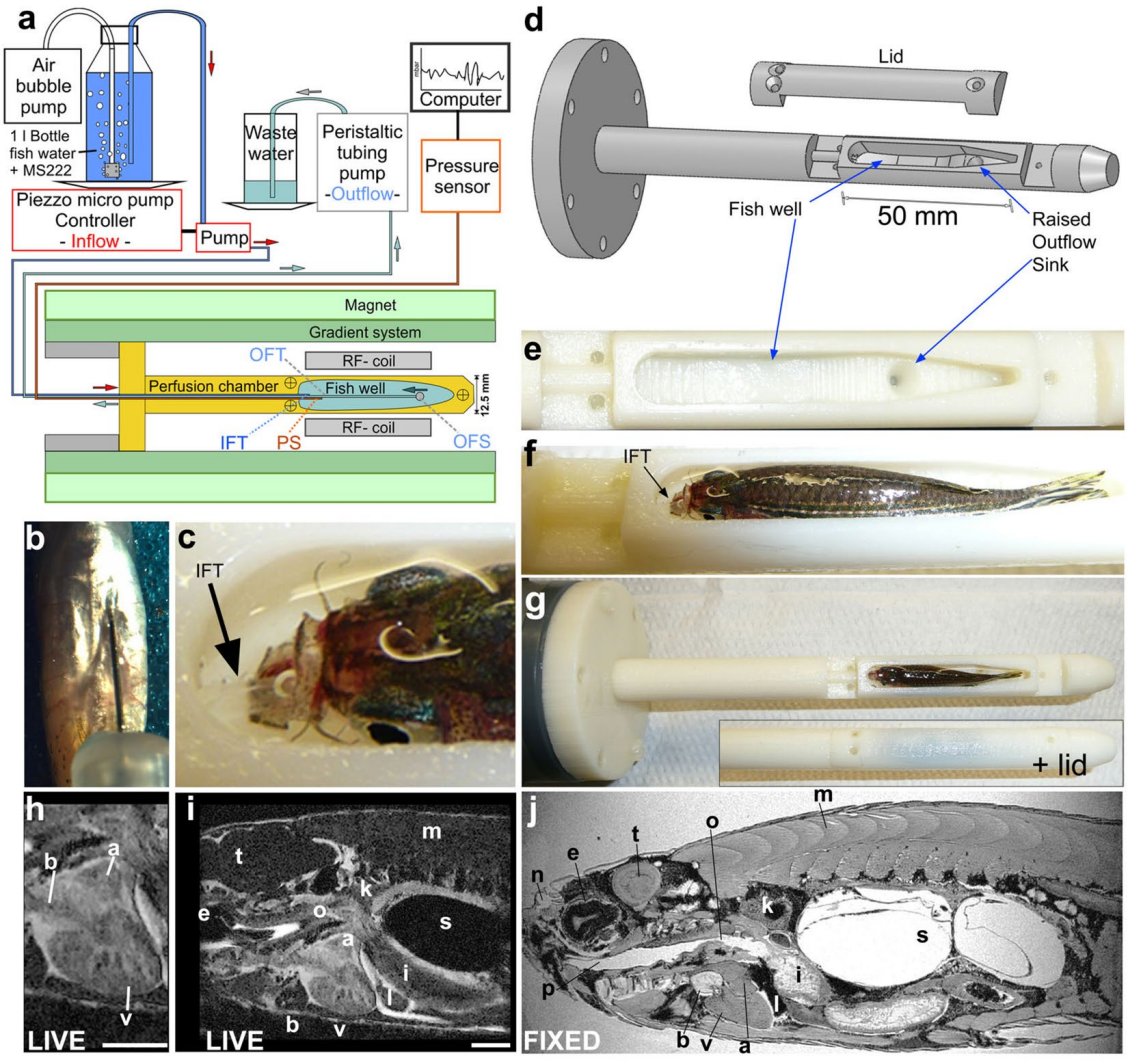
Respiratory and anaesthesia perfusion set-up for adult zebrafish *in vivo* imaging. **(a)** Schematic drawing of the perfusion system and MRI set-up. IFT – inflow tube, OFS – outflow sink, OFT – outflow tube, PS - pressure sensor **(b)** Ventral view (anterior at the top) of a zebrafish showing an intra-peritoneal injection of contrast agent prior to MRI. **(c)** Photograph showing the thin transparent respiratory perfusion inflow tube (IFT, arrow) positioned in the zebrafish mouth. Note the water level is above the eyes of the fish and covers the gills. **(d)** Drawing of the fish perfusion chamber as used for 3D printing illustrating the contours of the fish well. **(e)** Empty fish well of the perfusion chamber (view from top) with the outflow sink visible. **(f)** Fish well containing an adult zebrafish with perfusion inflow tube (IFT, arrow) in its mouth ready for imaging (see also c). Note the fish is 2/3 covered with water and eyes and gills are submerged. **(g)** Whole perfusion chamber (top view) containing a fish with and without the lid. The perfusion chamber is screwed onto a larger tube (dark grey) to insert it into the MR magnet. **(h–i)** Single image plane of a 3D image stack of a live adult zebrafish heart (h) or the whole scan field (i) from eye to mid swim bladder in (sagittal view). Scale bar 1mm. **(j)** Single image plane of a 3D image stack of a paraformaldehyde-fixed adult zebrafish in sagittal view. a-atrium, b-bulbus arteriosus, e-eye, i-intestine, k-kidney, l-liver, m-muscle, n-olfactory pit, o-oesophagus, p-pharynx, s-swim bladder, t-tectum opticum, v-ventricle.

To accommodate adult zebrafish for MRI and to maximize image resolution, we built a miniaturized (outer diameter 12.5 mm) water and anaesthesia perfusion set-up to hold a live fish in a natural body position and respirated for extended periods of time (Fig. 2). We further established a fast, Compressed Sensing (CS) accelerated high-resolution gradient echo MRI protocol to reliably characterize the lesion in live specimens that had been injected with a paramagnetic (Gadolinium-based) MRI contrast agent prior to scanning (Fig. 2 b). With this set-up we were able to scan live adult zebrafish under anaesthesia (and thus immobilized) and physiological conditions (upright in water and with gills perfused with water) for several hours and with 100% recovery rate and were thus able to image the same fish repeatedly during the repair process, which can last many weeks. The aim was to display the whole cardiac morphology and in particular the ventricular injury with the highest resolution possible, without resolving cardiac motion. We optimised the approach so that each scan lasted ~30 min (see data acquisition in Methods) covering a volume of 8 × 8 × 8 mm through the thoracic region of each fish at 31 µm isotropic voxel resolution. Each fish could then be recovered, transferred to the aquarium and be re-imaged on subsequent days during the repair process.

**Figure 3:**
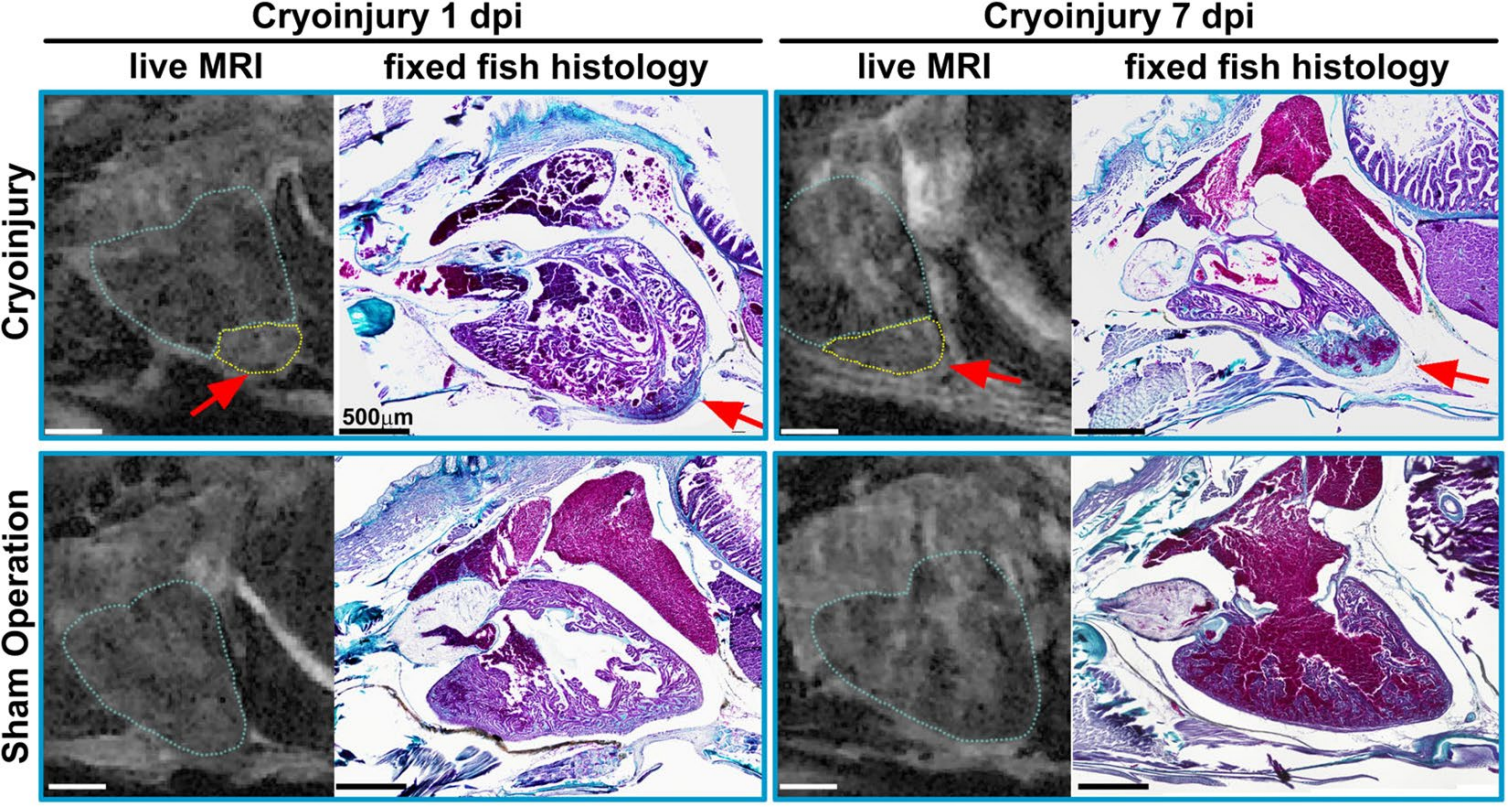
Confirming *in vivo* MRI with histology: MR images of the live ventricle in two cryo-injured (see arrows and outlined in yellow) and two sham operated wt fish at 1 and 7 days post injury and their corresponding histological sections. To confirm proper identification of the injury, the fish were sacrificed and fixed for histology within minutes after the *in vivo* scan. Trichrome staining was used to highlight the injured region in the same heart; collagen appears blue (highlighting the injury region) and cytoplasm pink. n = 2 per group. Scale bar 0.5 mm.

**Figure 4:**
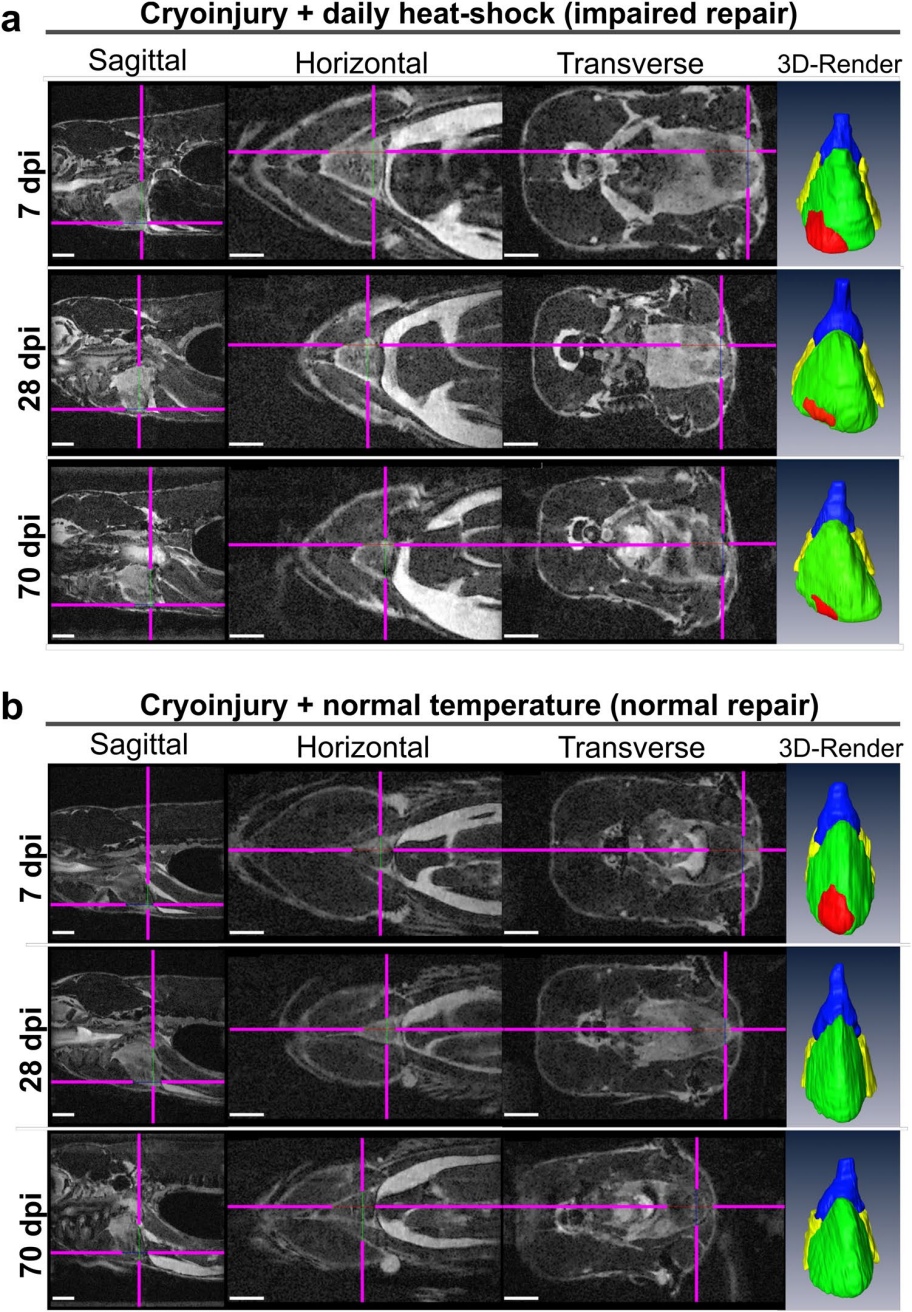
Impaired heart regeneration in a live specimen can be followed for at least 70 days. Orthogonal views of MR images of the two cryo-injured live *Tg*(*hsp70l:dnfgfr1a-EGFP*) +/− fish with **(a)** and without **(b)** daily heat-shock (HS) treatment at 7, 28, and 70 days post injury. Shown is the precise location of the lesion in each fish in 3 optical planes (sagittal, horizontal and transverse) and as a ventral view 3D Amira reconstruction (3D Render) of the heart (red-lesion, green-ventricle, blue-bulbus arteriosus, yellow-atrium). The injury location is indicated in each plane by pink crosshair lines, in order not to obscure the heart, the cross-hairs are drawn thinner above the heart and injury site. n = 4 per group. Scale bar 1 mm.

**Figure 5:**
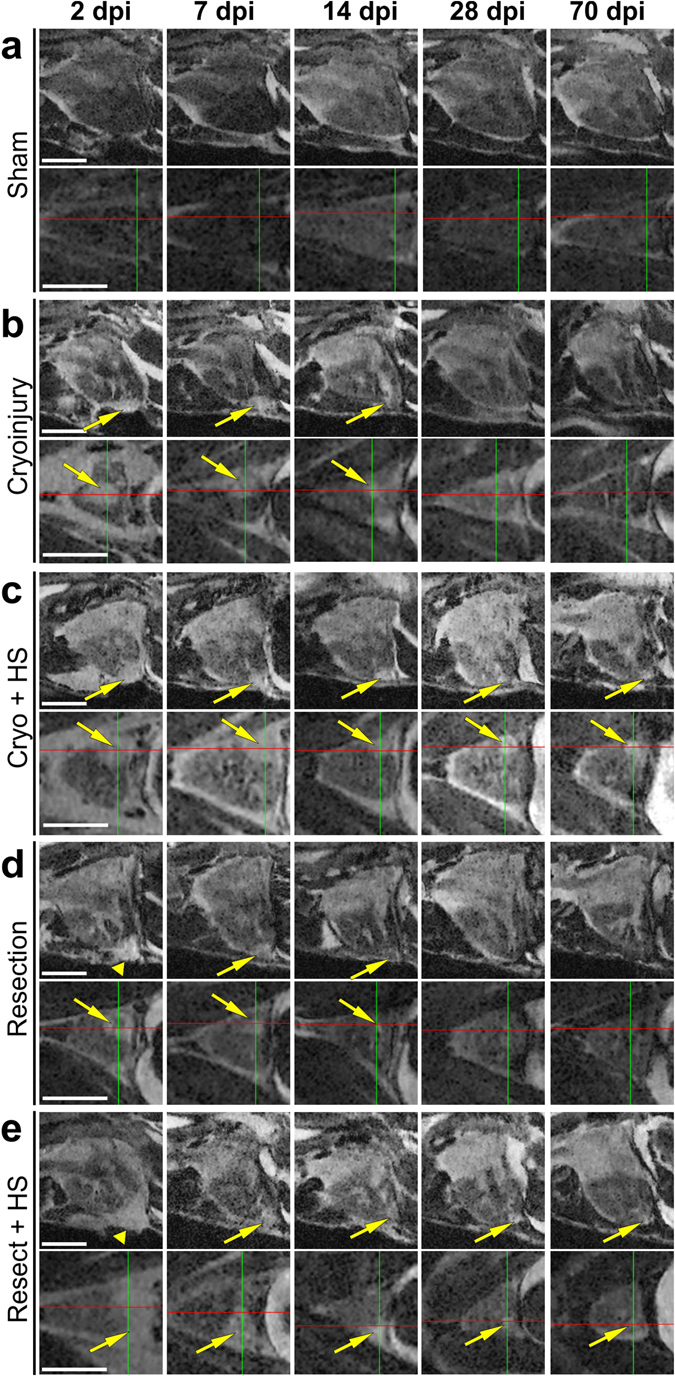
Comparing well and poorly healing hearts for 70 days post injury (dpi). **(a–e)** Optical sagittal and horizontal MR sections of 5 individual *Tg*(*hsp70l:dnfgfr1a-EGFP*) +/− adult fish imaged repeatedly over 70 days showing **(a)** a sham control ventricle (no injury but opened pericardium), and the regeneration process in **(b)** cryo-injured and **(d)** resected ventricles, and **(c)** cryo-injured plus heat-shock (HS) and **(e)** resected plus HS treated ventricles at 2, 7, 14, 28, and 70 days post injury (dpi). **b + d** serve as injured but not HS treated controls to visualise normal versus impaired (**c + e**) heart repair. Arrows point to the injury location and arrowheads indicate the blood clot following resection injury. Scale bar 1 mm.

To confirm that we could indeed successfully identify cardiac injury with MRI *in vivo*, we scanned sham operated and cryo-injured wild-type fish either at 1 or 7 dpi, followed by immediate sacrificing, fixation and Trichrome histological staining. This allowed us to compare the images of the same heart injury/fish obtained non-invasively from *in vivo* MRI with their corresponding *ex vivo* fixed and microtome cut histological sections (Fig. 3). The *in vivo* MR images presented the lesion as brighter (hyper-intense) than the uninjured adjacent myocardium, indicating contrast agent enrichment (for comparison see also Suppl. Movies 1–4, 7, 8 and Figs 4, 5). We found that the bright region identified as the lesion in the MR image matched well to the location of the Trichrome-stained histological sections that is stained bluish and classically is associated of being collagen rich of the same cryo-injured ventricles both at 1 and 7 dpi (Fig. 3). Although Trichrome staining is a good indicator of collagen rich tissues and is frequently used in cardiology studies to highlight scar regions after injury, collagen protein can currently only be unambiguously identified by antibody staining or via the use of transgenic animals that produce fluorescently labelled collagen protein. However, further support for the increase of various collagens in ventricles after cardiac injury can be derived from published data-sets on zebrafish heart repair ^15, 16^. The observed intensity contrast between intact and damaged tissue in live images was subtle, albeit distinctly notable on all optical planes covering the injury when moving through the entire 3D image stack (see Suppl. Movies 3, 4, 7, 8). 3D orthogonal views were derived to accurately define the cardiac lesions in live specimens and visualize heart regeneration (see Suppl. Movies 2, 4 and 8). Taken together, the developed MRI method allowed us to distinguish between healthy and damaged heart muscle and identify cardiac cryoinjury with confidence in living ~one year old zebrafish.

### MRI is suitable to discriminate between normal and impaired healing of cardiac injuries in adult zebrafish *in vivo*

We next investigated if the technique was sensitive enough to allow detection of impaired healing, as could be envisaged in experiments to study gene functions during heart repair. To test whether it was possible to discriminate between different rates/efficiencies of cardiac regeneration with this method, we utilized heterozygote heat-shock (HS) inducible dominant negative *fgfr1a* fish (*Tg*(*hsp70l:dnfgfr1a-EGFP*)), which when heat-shocked daily, exhibit impaired heart regeneration following ventricular resection^17^ (Figs 4 and 5). We used uninjured and sham surgery (opened pericardium, but no cardiac injury), cryo- and resection injured fish and scanned each of them before and at 2, 7, 14, 28 and 70 dpi; with some of the cryo- and resection injured fish undergoing a daily HS treatment and the other groups serving as controls. In both uninjured (data not shown) or sham operated *Tg(hsp70l:dnfgfr1a-EGFP)* fish, no hyper-intense bright region was observable at the ventricular apex (Fig. 5a and Suppl. Movies 1 and 2). HS treated cryo-injured (Fig. 5c, Suppl. Movies 5 and 6) or resected (Fig. 5e, Suppl. Movies 9 and 10) fish were initially indistinguishable from non-HS transgenic cryo- or resection- injured controls (Fig. 5b,d, Suppl. Movies 3,4,7 and 8), with the lesion sites clearly visible across all four groups (yellow arrows). In cryo-injured hearts the lesion was evident as a brighter (hyper-intense) region compared to the uninjured adjacent myocardium throughout the regeneration period (Fig. 4, Fig. 5b,c and Suppl. Movies 1 and 3). In resection injured fish, the initial large clot that formed at the ventricular amputated apex (clearly visible as a hyper-intense region, see Fig. 5d,e and Suppl. Movie 8) decreased rapidly in size with time. From 7 dpi onwards a bright hyper-intense area that was smaller than comparable apexes of cryo-injured hearts was visible in resection injured ventricles (see arrows, Fig. 5). At 28 dpi small bright regions were still visible at the injured ventricular apex of the HS groups (Fig. 4, Fig. 5c,e; see crosshairs and arrows), indicating impaired regeneration. At 70 dpi, most notably in cryo-injured ventricles of heat-shock treated transgenic fish, these discrete bright (hyper-intense) regions were still evident (Fig. 4); the resected heat-shock treated ventricles had comparatively smaller bright regions at the equivalent time-point (Fig. 5c,e). Taken together, both cryo-injured and resection-injured ventricles in heat-shock treated *Tg(hsp70l:dnfgfr1a-EGFP)* fish revealed impaired heart regeneration, when compared to non-heat-shocked transgenic injured controls, in agreement with previous histological analyses^1, 9, 11, 13, 17^. We conclude therefore, that the presented MRI method is suitable to image heart regeneration, non-invasively and under optimal physiological experimental conditions, in live adult zebrafish with sufficient spatial resolution to discriminate small histological changes in scarring and tissue restoration.

## Discussion

MRI has long been a mainstay of *in vivo* imaging in the clinical context, but also in mammalian animal models of human disease, providing anatomical, functional and metabolic information, whilst enabling complete recovery after the scan procedure. However, MRI has been challenging for small structures and small model organisms, such as zebrafish, due to the inherent lack of sensitivity of the technique, resulting in limited spatial resolution. In order to complement the current set of experimental platforms with which to study heart repair mechanisms in the adult zebrafish, we set out to develop an MRI technique that can be applied to such small hearts with ~750 µm ventricle diameter. To successfully resolve anatomical differences in these small structures required miniaturisation of the set-up allowing the use of powerful pre-clinical high-field strength magnets in combination with an RF coil optimized in diameter and magnetic properties. To the best of our knowledge, our study presents the first high-resolution, non-invasive, cardiac imaging technique for adult (non-transparent) zebrafish with which heart repair/regeneration can be imaged longitudinally in live fish. To date, assessment of myocardial injury has been mostly carried out using destructive histology, which provides high spatial resolution images of tissue sections from which scarring can be estimated. However, this necessitates a separate cohort of animals for each time-point post injury and renders measurements of the initial injury in any individual impossible, with caveats in data interpretation due to inherent variation in the extent of injury and the downstream response between individuals. Furthermore, histological analysis is time consuming, and also prevents accurate assessment of the injury due to tissue changes caused by fixation, dehydration and sectioning. *In vivo* MRI is non-destructive allowing initial assessment of wound extent and repeated measurement of each animal over the time course of the study to assess subsequent healing. This is particularly important for interventions aiming at experimentally modulating regeneration, which ultimately requires normalization to the initial degree of injury.

Our data demonstrate that high resolution MRI can be used to accurately visualize cardiac lesions in zebrafish for *in vivo* studies, and that the method is sufficiently sensitive to distinguish between good and poorly regenerating cardiac tissue over long periods of time. Whilst recent studies using Echocardiography^4^ and invasive Doppler Flow^18^ Imaging have provided some functional insight into the heart repair process, both methods lack sufficient spatial resolution to accurately determine tissue restoration. Moreover, they were carried out under physiologically sub-optimal conditions with fish immobilized upside down and without respiratory perfusion, thereby adversely affecting baseline cardiac physiology. In a previous study^3^ that demonstrated the first live zebrafish MRI images, an *ex vivo* (fixed tissue) isotropic image resolution of 137 μm and an *in vivo* resolution of 78 μm × 78 μm × 200 μm (2D RARE pulse sequence) was achieved with their set-up. We have made a number of improvements over the work presented by Kabli *et al*.^3^. By developing a miniaturized perfusion set-up, we were able to use a smaller RF coil, which was better optimized to the geometry of the fish resulting in improvements in signal to noise in the images. By applying a compressed sensing-based acquisition, we were able to substantially reduce the scan time required for the images, improving signal-to-noise per unit time. Consequently we were able to achieve an isotropic resolution of 31 μm in the live zebrafish. This is a 40-fold improvement in spatial resolution compared to the previous study. In a zebrafish ventricle that is ~750 μm wide, this substantial increase in spatial resolution finally makes MR imaging suitable to visualize even small tissue changes such as in ventricular wall lesions, at close to cellular resolution.

*In vivo* MRI, therefore, offers the most advanced platform for high-resolution 3D live deep-tissue imaging and visual proof of adult zebrafish regeneration, overcoming the major drawback of tissue optical density during light microscopy. This study demonstrates that MRI in live adult fish can be employed in the same manner as for larger experimental models, such as rodents and pigs. An MRI scanner, now routinely used in many facilities for rodent imaging, can readily be adapted for *in vivo* fish imaging. We anticipate that adult zebrafish MRI may be used in conjunction with molecular and cellular approaches to dissect out mechanisms underlying heart (or other organ-based) regeneration. We believe that this technique will allow further investigations into scar resolution and cardiac remodelling at a previously unprecedented level of detail. In addition, the set-up is easily scalable and can be applied to other fish species (eg. medaka or cave fish) that are emerging as novel model organisms to study heart repair, as they have different regenerative responses after cardiac injury^19^. Furthermore, the method described is not restricted to cardiac imaging and can be readily applied for non-invasive imaging of other injured or diseased tissues, such as models of vessel, brain or spinal cord injury, tumour formation or fat distribution, consequently expanding the experimental range of the zebrafish model and representing a valuable resource for the biomedical research community.

## Methods

### Zebrafish strains and husbandry

Adult wildtype (wt) (KCL strain) and *Tg*(*hsp70l:dnfgfr1a-EGFP*)^20^ 1-2 year old zebrafish were used in batches from the same spawning, so that animal size and age were matched as much as possible to reduce variability. Fish were housed in a Techniplast aquarium system [28 °C, 14/10 hours light/dark cycle, fed 3x daily with dry food and brine shrimp]. Sham operated and injured fish were kept in individual tanks throughout the entire experiment to identify each individual fish. For MR imaging the fish were transported to the MR suite in a carrier box containing individual small tanks (one 850 ml Lock & Lock rectangular container per fish). At the MRI suite, fish were housed at 22 °C in a designated animal room, kept overnight and returned to the isolation room in the aquarium on the following day.

### Cardiac surgery

All procedures/protocols were carried out in accordance with British Home Office regulations, under project licenses held in the contributing labs and having been approved by Home office inspectors and local representatives. Zebrafish cryoinjury of the ventricle was performed as previously reported^10^. For resection injury^1^, the initial steps were performed as for the cryoinjury, but after exposing the heart ~15% of the ventricular apex was amputated using spring scissors (Fine Science Tools, FST No. 15003-08) and fish were not agitated with a pipette during recovery to prevent reopening of the forming blood clot. Sham surgery involved all steps routinely done before cryo- or resection injury (from anaesthesia in 160 mg/l MS222 (Sigma) in fish-water to drying the apex of the exposed ventricle with a small piece of tissue), then the ventricular apex was briefly touched with a metal probe at room temperature, followed by the normal recovery in a tank without anaesthetic.

### Daily heat-shock treatment

Heat-shock treatment (HS) of Tg(*hsp70l:dnfgfr1-EGFP*) fish was performed daily either in the aquarium (thermostat) or at the MRI suite (water bath). Generally the HS group fish were kept in small individual perforated tanks within a large 45 litre tank, and the heat-shock procedure was carried out via a programmable thermostat (Lauda Eco Gold). The temperature was raised to 38 °C within 30 min (with a tolerance level of 0.4 °C) and then the tank was left to cool down to normal aquarium temperature, which took several hours (temperature curves recorded by thermostat). It should be noted that heat-shock treatment resulted in a significantly increased rate of respiration for the fish, and thus the rate of opercular movement increased to a level similar to intense exercise as in swim tunnel performance challenges. During the course of the experiment, in the resection + HS group, 1 fish died at 2 dpi and 2 at 7 dpi, whereas in the cryoinjury + HS group 2 fish died at 7dpi and 2 at 40 dpi. At the MRI suite, individual fish were heat shocked in small tanks that were immersed in a transportable water bath (rising from 22 °C to 38 °C within 45 min, keeping 38 °C for 1 h and left to cool down to 22 °C).

### Animal holder and perfusion set-up design

In order to maximize scan resolution and prevent any potential water leakage within the MR scanner, we designed a miniature perfusion set-up to keep adult zebrafish alive under physiological conditions inside the magnet, allowing for extended imaging periods. The perfusion chamber was designed with an inflow tube that is placed into the mouth of the fish, and a raised outflow hole in the chamber bottom at the tail end of the fish. This ensured that the fish remained upright and sitting in a reservoir of water, and that water flows out from both its gills ensuring proper perfusion and maintaining a water coating over the fish.

The animal holder was made from thermoplastic ABS (acrylonitrile butadiene styrene) using an HP Designjet 3D-printer and SolidWorks (CAD package) software was used to draw, optimize and print the chamber. This 3D printing enabled the creation of a perfusion chamber with minimal space requirements to fit into the RF-coil with an inner diameter of 13 mm. The chamber had separate water inflow and outflow tubes (see Fig. 2). Through the upper tube, an inflow perfusion PE tube (outer diameter 0.8 mm) and a fibre optic pressure sensor fibre (Samba sensor, Harvard Apparatus) were fed into the fish chamber. While the water inflow tube was placed into the fish’s mouth (see Fig. 2c), the pressure sensor tip was placed underneath the fish. The pressure sensor was connected to a Powerlab Chart Recorder (AD Instruments) and the signal was analysed using Chart5 software. The pressure sensor was used to monitor fish well water levels and allowed us to remotely monitor chamber water perfusion to ensure the fish’s welfare while in the scanner. A second tube (silicone) was fitted into the outflow opening with a nylon adapter nozzle, connecting the outflow tubing with the chamber drainage system, a thin tunnel running underneath the fish chamber to connect with the raised outflow sink hole at the rear end of the fish chamber. The inflow and outflow tubes and the pressure sensor were sealed and secured at the chamber end with silicon grease, and in order to prevent any leakages during MR imaging the outside of the perfusion chamber was coated with silicon grease too before water perfusion commenced. The perfusion set-up required three pumps (see Fig. 2): a standard aquarium bubble pump that oxygenated the water; a piezzo-driven micro-pump (Bartels, mp6 micro-pump) that provided the fish perfusion with a low flow rate adjustable to between 0.5-5 ml/min pumping water from the bottle into the mouth of the fish; and a peristaltic tubing pump (Gilson Minipuls) that pumped water through the outflow tube from the perfusion chamber into the waste beaker. All components were assembled outside the magnet room and fed through a waveguide, leaving all equipment in the prep room and only the animal holder was brought into the MR room. All tubing and cable optic wiring had to be of sufficient length, such that the perfusion chamber could be inserted into the isocentre of the magnet. After confirming that the set-up was ready to use and that the pressure sensor was equilibrated, the anaesthesia-maintenance water inflow and outflow were calibrated (with stopwatch and scale) to reach the desired perfusion rate for the fish. Once the perfusion set-up was assembled and running steadily, change-over time between fish was <1 min.

### Fish anaesthesia perfusion for imaging

In order to keep the fish respirated and anaesthetized for the duration of the MR scan, the tube-fed respiratory flow perfusion was carried out with an anaesthetic concentration of 140 mg/l MS222 (Sigma) and Tris buffered in aquarium fish water. The minimum gill perfusion flow rate was calculated to be at least ~500 μl/g·min; a target flow rate of 1-2 ml/g/min was used for the anaesthetized fish to ensure efficient oxygenation even in the presence of less optimal perfusion of the gill branches. After testing prolonged perfusion times (up to 3 h) and two different rates (2 and 4 ml/g·min), no externally visible signs of distress or discoloration of the gills were observed; all fish recovered without damage and lived for several months with no subsequent problems. As high water perfusion rates could potentially lead to respiratory alkalosis and reduced heart rate, a flow rate of 1.2 ml/min for fish around 0.5 g (±0.2 g) and 1.8 ml/min for larger fish (~1 g) were used.

### Contrast agent injections, fish prepping for imaging and recovery procedure

Paramagnetic, Gadolinium-based MRI contrast agents are readily used to highlight the injured heart region. To prepare the fish for imaging, it was transferred (with a net) into an anaesthesia tank (MS222 160 mg/l) until the point at which opercular movement just ceased and the fish no longer responded to touch of the tail fin. The fish was then placed (manually and via coarse forceps) ventral side up on a moist slitted sponge (as for cardiac injury) and was injected intra-peritoneally (see Fig. 1b) with a small volume of 3 µl/100 mg body weight of 16.6 mmol/l [=0.25 µmol per 0.5 g fish] Omniscan (Gadodiamide, GE Healthcare) diluted in physiological saline solution, using a 30 G fine insulin syringe (BD micro-fine 0.3 ml U-100, 0.3 mm × 8 mm). For repeated MR imaging of the same fish in the time-lapse experiment, injections were alternated between the left and right side of the belly. After injection, the fish was transferred into a waiting perfusion chamber (same chamber type and perfusion set-up as for imaging) and the water inflow tube was inserted into its mouth using fine curved forceps (Dumont 7b). After ~10 min the fish was transferred into the imaging chamber, the lid was placed on top and sealed with silicon grease; then the perfusion chamber was inserted into the RF coil and positioned at the isocentre of the magnet for imaging. The use of a second separate perfusion chamber (plus corresponding pump) placed on the preparation bench reduced swap-over times between fish, allowing the fish to be fully anaesthetized prior to imaging, and ensuring that the contrast agent could distribute throughout the fish for 15 min prior to the start of the scan.

Following imaging, fish were removed from the chamber using coarse forceps and placed into the experimenter’s palm submerged in the recovery tank, where the fish was ventilated with anaesthetic-free aquarium water with a 3 ml plastic pipette until the fish started moving (see Movie 11). After recovery, the fish were monitored for 15 min in the recovery tank before being transferred back into a housing tank in the animal holding room. Recovered fish were subsequently monitored (~every 45 min) after the scan for the rest of the scan day and 3-4 times daily (during feeding) in between scans through the entire experimental duration.

### RF coils and MRI System

MR imaging was carried out using a preclinical 9.4 T (400 MHz) MR system (Agilent, Santa Clara, US), comprising a horizontal magnet (inner diameter (i.d.) = 210 mm), an actively shielded gradient system (1 T/m, rise time 130 µs, i.d. = 60 mm) and a DirectDrive^TM^ 2 console. A 13 mm i.d. quadrature-driven birdcage RF coil (Rapid Biomedical, Rimpar, Germany) was used to transmit and receive the MR-signals.

### Fixed specimen *(ex vivo)* scan procedures

Fish were fixed whole in 4% Paraformaldehyde in Phosphate buffered saline (PBS) at 4 °C for several days, washed twice with PBS and then incubated overnight with PBS + 2 mM Omniscan (Gadodiamide, GE Healthcare) (PBSo^+^) at 4 °C. Whole fish were embedded in 1% agarose in PBSo^+^ in an NMR tube (outer diameter: 13 mm – Flourochem, Hadfield, UK) and imaging was performed using a fast gradient echo sequence (TE/TR = 10/30 ms) as previously reported ^21^. Each *ex vivo* fixed specimen was scanned in ~9 h in unattended overnight runs at a near isotropic resolution of 25.4 × 25.4 × 26.0 µm. Our experimental rationale for this *ex-vivo* study was to have 3-4 animals per time point for both cryo and resection injury and two animals for sham controls. Unfortunately, and highlighting the variability of the injury procedures, one animal had no detectable cryoinjury and was thus excluded from Fig. 1B, and a further two animals died several days after cryoinjury, leaving only two fish for the 60 dpi time point. As we had one separate 7 dpi fish of the same batch, which we used to set up the scan parameters, volume calculations and equipment, we decided to include its data in our calculations.

### Data acquisition, scan routines and data and image processing

In order to scan the many fish of the various experimental groups, scan times in this study were as short as practical while maintaining a sufficiently high image resolution and signal to noise ratio. It was established that 2 × 15 min scans adequately achieved this balance between speed and image quality; it was also similar in time to that required to acquire a 3D confocal microscope scan as they are routinely performed in conventional zebrafish live imaging. Furthermore, it is also essential from an animal welfare point of view to minimize the anaesthetic burden (and therefore scan time), particularly for fish with heart injury.

For *in vivo* imaging, after positioning the perfusion chamber in the magnet, scouting was performed to localize the heart (2D gradient echo sequence, TE/TR = 2.7/60 ms, eight 0.3 mm sagittal slices, field-of-view (FOV) 20 × 12 mm, matrix size 512 × 128, 4 averages). The RF-coil was then manually tuned and matched to 50 Ω, and the magnetic field homogeneity automatically optimized (“shimming”). Following RF-pulse calibration, a fast, compressed sensing accelerated (acceleration factor = 2) 3D gradient echo sequence (TE/TR = 2/60 ms, FOV 8 × 8 × 8 mm, 7 mm sagittal slab, matrix size 256 × 256 × 256, 2 averages) was applied. The resulting experimental resolution was 31 × 31 × 31 µm, and the total scan time including preparation was <45 min. Raw image data were transferred to a workstation (Mac Pro, Apple Inc.) to perform compressed sensing reconstruction and image processing offline using purpose-written Matlab scripts (Matlab 2013a, Mathworks).

The images, ${\bf{I}}$, were reconstructed by solving the following equation:

1 $${{\bf{I}}}^{\ast }=\mathop{{\rm{a}}{\rm{r}}{\rm{g}}{\rm{m}}{\rm{i}}{\rm{n}}}\limits_{{\bf{I}}}\,{\parallel {\mathscr{F}}({\bf{I}})-{\bf{Y}}\parallel }_{2}^{2}+{\lambda }_{1}{\parallel \varphi (|{\bf{I}}|)\parallel }_{1}+{\lambda }_{2}{\parallel {\bf{I}}\parallel }_{{\rm{T}}{\rm{V}}}.$$

The first term in Eq. 1 controls for the data consistency between the acquired k-space data ${\bf{Y}}$ and the reconstructed image. ${\mathscr{F}}$ denotes the 3D Fourier transform. The second term controls the sparsity of the image under the transformation ${\rm{\varphi }}$. In this case, ${\rm{\varphi }}$ was a three level Daubechies wavelet transform applied to the image magnitude. Finally, the third term controls the total variation (TV) of the image, which was required to maintain image smoothness. ${\lambda }_{1}$ and ${\lambda }_{2}$ control the relative influence of the sparsity and TV terms, and were empirically chosen to be ${10}^{-4}$ and ${10}^{-5}$respectively. The reconstruction was performed using nonlinear conjugate gradient descent optimisation with 30 iterations. The compressed sensing reconstructed k-space data were zero-filled (factor of 2), and filtered (modified Butterworth filter) prior to Fourier transform. The magnitude images were analysed using Amira 3D Software (FEI^TM^, https://www.fei.com/software/amira-3d-for-life-sciences/). Briefly, 3D image stacks were imported into Amira, where the ventricle and lesion was manually segmented, and the objects were reconstructed and rendered to produce model images and volume measurements. Movies were made using either Fuji ^22^ for whole data stack exports or a purpose-written Matlab script for extracting image subsets based on a drawn mask (Matlab 2013a, Mathworks).

### Ethical statement

All experiments were carried out in compliance with the revised Animals (Scientific Procedures) Act 1986 in the UK and Directive 2010/63/EU in Europe with the appropriate Home Office license and have been approved by Oxford’s central Committee on Animal Care and Ethical Review (ACER).

## Electronic supplementary material

Suppl. Movie 1

Suppl. Movie 2

Suppl. Movie 3

Suppl. Movie 4

Suppl. Movie 5

Suppl. Movie 6

Suppl. Movie 7

Suppl. Movie 8

Suppl. Movie 9

Suppl. Movie 10

Suppl. Movie 11

Supplementary Movie Legends
