# Supplementary material for: High-Resolution Magnetic Resonance Imaging of the Regenerating Adult Zebrafish Heart
Source: Sci Rep. 2017 Jun 7;7:2917. doi: 10.1038/s41598-017-03050-y (PMC5462770; doi:10.1038/s41598-017-03050-y)
Supplement: Supplementary file 12 — Supplementary Movie Legends [file 41598_2017_3050_MOESM12_ESM.doc]

**High-Resolution Magnetic Resonance Imaging of the Regenerating Adult Zebrafish Heart**

Jana Koth^1,2,*^; Mahon L. Maguire^3^; Darryl McClymont^3^; Leonie Diffley^3^; Victoria L. Thornton^3^, John Beech^4^; Roger K. Patient^1^; Paul R. Riley^2^; Jürgen E. Schneider^3,*^.

^1^Weatherall Institute of Molecular Medicine, JR Hospital, Oxford OX3 9DS

^2^Department of Physiology, Anatomy and Genetics, Oxford OX1 3PT

^3^BHF Experimental MR Unit, Wellcome Trust Centre for Human Genetics, Oxford OX3 7BN

^4^Department of Oncology, Oxford OX3 7DQ

^1-4^Oxford University, Oxford, UK

**Supplementary Information File**

**Movie Legends**

**Movie 1:** **Live MR image stack of a zebrafish 2 days post sham operation control:** Example of a 3D *in vivo* MR image, showing the trunk region of a live adult *Tg*(*hsp70l:dnfgfr1a-EGFP*)+/- zebrafish with a sham operation (opened pericardium but no injury to the heart) at 2 days post sham injury procedure (dpi) with 31 μm isotropic voxel resolution. The movie runs through the individual optical planes of the 3D image stack in lateral view (sagittal sections) starting with the left body half. Anterior is to the left, dorsal at the top. The horizontal field of view (8 mm) is from mid-way through the eye on the left, to the first third of the swim bladder on the right, with the heart in the ventral middle of the image (see arrow). For further anatomical details see Fig. 2 h-j. The pericardial opening that exposes the ventricle to the water is only visible during the time the writing appears.

**Movie 2:** **MR time lapse image series in 3 view planes of a live zebrafish at 2, 7, 14, 28 and 70 days post sham operation control:** The image series shows in sagittal, transverse and horizontal views, an image subset which is centred on the heart region at 2, 7, 14, 28 and 70 days post sham operation in a *Tg*(*hsp70l:dnfgfr1a-EGFP*)+/- adult fish (surgery control).

**Movie 3:** **Live MR image of a zebrafish 2 days post cryoinjury**: Example of a 3D *in vivo* MR image, showing the trunk region of a live adult *Tg*(*hsp70l:dnfgfr1a-EGFP*)+/- fish with a cryo-injured heart at 2 days post injury (dpi) with 31 μm isotropic voxel resolution. The movie runs through the individual optical planes of the 3D image stack in lateral view (sagittal sections) starting with the left body half. The horizontal field of view (8 mm) is from mid-way through the eye on the left, to the first third of the swim bladder on the right, with the heart in the ventral middle of the image. The cryoinjury (bright hyper-intense apex area) is indicated by a red dot. Anterior is to the left, dorsal at the top. For further anatomical details see Fig. 2 h-j.

**Movie 4:** **MR time lapse image series in 3 view planes of a live zebrafish at 2, 7, 14, 28 and 70 days post cryoinjury:** The image series shows in sagittal, transverse and horizontal views, an image subset which is centred on the heart region (injury indicated by red dot) at 2, 7, 14, 28 and 70 days post cryoinjury in a *Tg*(*hsp70l:dnfgfr1a-EGFP*)+/- adult fish (injury but no heat-shock).

**Movie 5:** **Live MR image of a zebrafish 2 days post cryoinjury + HS**: Example of a 3D *in vivo* MR image, showing the trunk region of a live adult *Tg*(*hsp70l:dnfgfr1a-EGFP*)+/- fish with a cryo-injured heart (plus daily HS) at 2 days post injury (dpi) with 31 μm isotropic voxel resolution. The movie runs through the individual optical planes of the 3D image stack in lateral view (sagittal sections) starting with the left body half. Anterior is to the left, dorsal at the top. The horizontal field of view (8 mm) is from mid-way through the eye on the left, to the first third of the swim bladder on the right, with the heart in the ventral middle of the image. The cryoinjury (bright hyper-intense apex area) is indicated by a red dot. Anterior is to the left, dorsal at the top. For further anatomical details see Fig. 2 h-j.

**Movie 6:** **MR time lapse image series in 3 view planes of a live zebrafish at 2, 7, 14, 28 and 70 days post cryoinjury + HS:** The image series shows in sagittal, transverse and horizontal views, an image subset which is centred on the on the heart region (injury indicated by red dot) at 2, 7, 14, 28 and 70 days post cryoinjury in a *Tg*(*hsp70l:dnfgfr1a-EGFP*)+/- adult fish + daily HS treatment (injury + HS).

**Movie 7: Live MR image of a zebrafish 2 days post resection injury**: Example of a 3D *in vivo* MR image, showing the trunk region of a live adult *Tg*(*hsp70l:dnfgfr1a-EGFP*)+/- fish with a resection injured heart at 2 days post injury (dpi) with 31 μm isotropic voxel resolution. The movie runs through the individual optical planes of the 3D image stack in lateral view (sagittal sections) starting with the left body half. Anterior is to the left, dorsal at the top. The horizontal field of view (8 mm) is from mid-way through the eye on the left, to the first third of the swim bladder on the right, with the heart in the ventral middle of the image. The blood clot (bright hyper-intense region at the pericardial opening) is indicated by a red dot. Anterior is to the left, dorsal at the top. For further anatomical details see Fig. 2 h-j.

**Movie 8:** **MR time lapse image series in 3 view planes of a live zebrafish at 2, 7, 14, 28 and 70 days post resection injury:** The image series shows in sagittal, transverse and horizontal views, an image subset which is centred on the heart region (injury indicated by red dot) at 2, 7, 14, 28 and 70 days post resection injury in a *Tg*(*hsp70l:dnfgfr1a-EGFP*)+/- adult fish (resection but no HS).

**Movie 9: Live MR image of a zebrafish 2 days post resection injury + HS**: Example of a 3D *in vivo* MR image, showing the trunk region of a live adult *Tg*(*hsp70l:dnfgfr1a-EGFP*)+/- fish with a resection injured heart (plus daily heat shock) at 2 days post injury (dpi) with 31 μm isotropic voxel resolution. The movie runs through the individual optical planes of the 3D image stack in lateral view (sagittal sections) starting with the left body half. Anterior is to the left, dorsal at the top. The horizontal field of view (8 mm) is from mid-way through the eye on the left, to the first third of the swim bladder on the right, with the heart in the ventral middle of the image. The blood clot (bright hyper-intense region at the pericardial opening) is indicated by a red dot. Anterior is to the left, dorsal at the top. For further anatomical details see Fig. 2 h-j.

**Movie 10:** **MR time lapse image series in 3 view planes of a live zebrafish at 2, 7, 14, 28 and 70 days post resection injury + HS:** The image series shows in sagittal, transverse and horizontal views, an image subset which is centred on the heart region (injury indicated by red dot) at 2, 7, 14, 28 and 70 days post sham operation in a *Tg*(*hsp70l:dnfgfr1a-EGFP*)+/- + daily HS treatment (injury + HS).

**Movie 11:** **Manual ventilation procedure for fast recovery of adult zebrafish after deep and prolonged anaesthesia**. After prolonged deep anaesthesia manual ventilation is required for recovery, allowing the fish to restart breathing on its own. If the pipette is held correctly during manual ventilation, the produced water stream will (drop) open the jaw of the anaesthetized fish and water will flow through the gills. This manual ventilation was maintained until the fish moved and began breathing independently again.
